# Fracture of the Patella

**Published:** 1912-09

**Authors:** W. L. Cooke

**Affiliations:** Columbus, Ga.


### FRACTURE OF THE PATELLA.

By W. L. Cooke, M. D., Columbus, Ga.

Fracture of the patella is not a very common injury, although it is more frequent than one would suppose, constituting about 2% of all fractures; but the dire results which are gotten in improperly treated cases, have prompted me to bring this subject up for your consideration.

The injury is usually caused by direct violence, muscular action, or a combination of the two, the latter being the most frequent cause. The fracture may be transverse, oblique, stellate, or longitudinal, the transverse being the most common variety, as this is usually the result of muscular action combined with direct violence.

The most common symptoms are pain, swelling and inability to extend the limb; crepitus is not usually elicited on account of the swelling and separation of the fragments. The fragments can often be palpated with an interval between them.

However, the diagnosis being simple, the treatment is what interests us most.

There are two methods of treatment.

The first, no matter how careful we may be in carrying it out, almost never gives a perfect result, and sometimes after this method, the patient is a cripple for life.

The results which are gained by the second method are almost certain to be eminently satisfactory, provided the technic is correct. As a preliminary to any form of treatment, an ice-bag should be applied to the knee for twenty-four to forty-eight hours in an effort to prevent as much as possible the serous effusion and hemorrhage into the joint, which is usually very great. Another way to partially prevent this, is to use pressure judiciously, in the way of sea sponges applied over the joint and held in place by a roller bandage.

The first method of treatment, the older one, which I do not advocate at all, except in certain cases of longitudinal fracture, is effected by means of a posterior splint of some kind, and loops of adhesive plaster fastened above the upper fragment and below the lower one, in such a manner as to draw them as near into apposition as possible. This method is usually attended with very indifferent results, the reasons for which, I think, are the following:

First, when any muscle has its point of attachment severed, it is bound to rectract, and become very much shorter; thus the quadriceps extensor retracts and pulls the upper fragment away from the lower. And it is very difficult, by means of any external force, to overcome this shortening.

In the second place, the large amount of clotted blood which collects in the joint, effectually separates the fragments, and is a long time being absorbed if left to nature. Then, again, the fibres of the quadriceps extensor tendon, which is attached over the anterior surface of the patella, fall down between the fragments and prevent bony union. Also, the upper fragment is usually tilted upward and forward so that its articular surface presents downward instead of backward.

Lastly, a point which is often neglected in treating these cases, is the fact that the lateral aponeurosis of the knee which is formed by a blending together of the fascialata and the lateral portion of the extensor tendon is usually quite badly torn, and this will not unite properly unless sutured together.

The plan of treatment which I follow is best outlined by the following case report:

On the night of December 24, 1911, I was called to see Mr. H., a powerfully built young man, thirty-five years of age. I found a transverse fracture of the right patella. The knee was swollen to about twice its natural size, quite painful and tender to pressure; the fragments were about an inch apart and could be easily palpated. I learned that the patient, in attempting to leave a room where he and some friends were having a drunken carousal, was met at the door by some one entering, who pushed the door open violently in his face. He threw up his knee to ward off the blow, and caught the full force of it on the patella while the knee was flexed, thus bringing into play the usual cause of direct violence combined with muscular action.

I put an ice-cap on the knee and kept it there about forty-eight hours, after which I used hot applications, the heat being more pleasant to the patient, for about one week. All the while I kept the limb on a posterior splint, but made no effort to bring fragments together, explaining to the patient that I proposed to operate later and sew the fragments together.

And just here I wish to explain my reason for delaying the operation:

If the operation were done immediately after the injury, we would be bothered by an annoying hemorrhage from the fractured ends of the bone, and torn ends of aponeurosis. Then, too, at this time we would be very liable to get a reaccumulation of clotted blood in the joint—a condition which is very desirable to avoid if possible, and by waiting about a week, we avoid this difficulty.

After sterilizing the field of operation with tincture iodine, I outlined a U-shaped incision, each end of which was over the condyle, well out to the side, and one-half inch below the line of fracture, the convexity of the U being slightly above the upper fragment. After infiltrating this line of incision with one-tenth per cent. cocaine solution as an anaesthetic, I turned down the flap, exposing both fragments, and evacuated a large amount of clotted blood from the joint, the whole joint cavity being full. I next trimmed off all torn fragment of soft tissue. I then began at one angle of tear in aponeurosis and sutured all the torn structures together, using very heavy forty-day chromic cat-gut. The sutures of the patella itself went only through the fibrous tissue and periosteum, and not into the bone. The skin wound was then closed, and a gauze dressing applied. The limb was put upon a posterior splint and left for ten days, when skin stitches were removed. Healing was my first intention, so I put the limb up in Plaster Paris and left it for six weeks, at which time union was solid. I then instituted massage and passive motion, letting patient get out on crutches, which he used for about two months. He now walks without any limp and is entirely well.

